# Comparative Study of Care Home Referrals During Three National COVID-19 Pandemic Lockdowns

**DOI:** 10.1192/bjo.2022.173

**Published:** 2022-06-20

**Authors:** Vellingiri Raja Badrakalimuthu

**Affiliations:** Surrey & Borders Mental Health Partnership NHS Foundation Trust, Guildford, United Kingdom

## Abstract

**Aims:**

To compare characteristics, presentation and treatment of care home patients referred to care home pathway team during three lockdowns.

**Methods:**

Data were collected from referrals to G&W care home pathway team during lock downs:

First: 23rd March 2020 to 30th June 2020

Second: 5th November 2020 to 2nd December 2020

Third: 5th January 2021 to 8th March 2021

Variables collected included number of referrals, age, gender, type of care home, reason for referral, type of behavioural and psychological symptoms of dementia (BPSD), diagnosis, new diagnosis of dementia, comorbidity, type and professional to make initial contact, blood tests at point of referral, appointments, duration on caseload, type of interventions for BPSD, admission, and use of antipsychotics. They were analysed for statistical significance at p value <0.05.

**Results:**

There were 23, 21 and 34 referrals respectively in the three lockdowns, with significant reduction in the weekly average of referrals (1.6), and number of men (17.4%) referred in the first lockdown. Significantly greater proportion of referrals in first lockdown was for BPSD (65.2%), with aggression (40%) as most common BPSD. Alzheimer's dementia was commonest dementia (67%) across lockdowns with fewer new diagnosis (21.7%) made in first lockdown. There was lower rate of delirium (21.7%) in fist lockdown associated with fewer blood investigations (56.5%) at point of referral. Although there was no difference by type of professional, number of appointments, and discharges, duration on caseload (median 58.5 days) was significantly longer during first lockdown. There was access to medical, nursing, and psychological therapies input during all lockdowns. There was reduction in medication prescription including antipsychotics (33%), with no new antipsychotics commenced in all lockdowns.

**Conclusion:**

Despite availability of mental health services, this study highlights reduction in access to mental health services as well as physical health investigations for elderly residents in care homes during the first lockdown.

